# American Academy of Dental Science

**Published:** 1890-10

**Authors:** 

**Affiliations:** American Academy Dental Science


### AMERICAN ACADEMY OF DENTAL SCIENCE.

The American Academy of Dental Science held its regular monthly meeting April 2, 1890, at the Boston Medical Library Association rooms. President F. N. Seabury in the chair.

Dr. H. A. Baker read a paper on "The Use of a Matrix in contouring Fillings." (For Dr. Baker's paper see page 608.)

### DISCUSSION ON DR. H. A. BAKER'S PAPER.

*Dr. Meriam.*—I believe, Mr. President, I am limited to the presentation of new forms, but I think there are hardly any really new forms to-day. We have practically two,—a band encircling the teeth, or a plate held against the teeth by a wedge. The Baker matrix certainly comes between the two. It has the value of the band matrix, with also the advantage that it does not curl away from the teeth at the cervical wall, as do the band matrices that require, in addition to the ordinary tightening by a screw, a wedge to hold them against the teeth at that point. I have always regretted that Dr. Baker omitted to publish a description of his matrix, that it might bear the date of its introduction. In these days when companies are forming on every side of us,—when we know not what dental meeting may contain a spy, or what invention may be taken from the profession,—these matters are of importance. We should not publish our inventions merely for the fame and honor of the thing, but that the profession should be able at least to give some history, some source, from which our instruments spring. I know the claim is made that the instrument-case is the real birthplace of the profession; but, gentlemen, in spite of all the practitioners who are ready to echo that sentiment, I think that it is hardly true. Dr. Baker's matrix should have been given a name, and all the various forms that have grown from it should have been called a modification or improvement of the Baker matrix. I meet the idea in all parts of the country, but I have rarely ever heard that Dr. Baker was the inventor, and I have often been obliged to show that he was.

I will present, first, some forms that were sent me by Mr. George Brunton, of Leeds, England, who offers a matrix that is tightened with a screw clamp, and also one that tightens with a screw wedge, or wedge clamp. These two forms I have here. They were sent me by Mr. Brunton through the courtesy of Dr. St. George Elliott, who was coming to this country. He also sent some to the Dental Schools of Harvard University and the University of Michigan, and, as some of the instructors of the former school are present, they can speak of their merits more intelligently than I am able to do. I think his key or wrench is the best I have ever seen, for, instead of the usual unsteady ones which we have, this has a guide, and by removing the ring it can be set at a different angle.

The matrix I prefer, in case only one wall is to be supplied, is a simple piece of steel, thick or thin, according to the distance between the teeth. For shaping the steel, the ordinary optician pliers (Stubbs's) gives a good form for bicuspids. The rounder pair of the French make (Tissol's) gives a better form for some teeth. Cold-rolled steel, with its evenness of working, the various thicknesses that are offered, and the ability to forge it somewhat, even when cold, into the shape we desire, is the most valuable. It is rolled to the thinness of five-thousandths of an inch in this country, and the French and Swiss watchmakers roll it down very much thinner. It can be procured from Goodnow & Wightman, Boston, Mass.; Wilkinson & Co., 184 Washington Street, Boston, Mass.; Chandler & Farquhar, 177 Washington Street, Boston, Mass.; Montgomery & Co., 103 Fulton Street, New York City; Frasse & Co., 92 Park Row, New York City; Peter A. Frasse, 95 Fulton Street, New York City; Niagara Stamping and Tool Company, Superior, corner Randall Street, Buffalo, N. Y.; Palmer, Cunningham & Co., 607 Market Street, Philadelphia; Simmons Hardware Company, St. Louis, Mo.; H. A. Pickering & Co., Cincinnati, Ohio.; Charles Strelinger & Co., 86 Woodward Avenue, Detroit, Mich.; Besley & Co., 175 Lake Street, Chicago, Ill.; Justinian Caire, 521 Market Street, San Francisco, Cal.; E. L. Parker & Co., 301, 303 South Charles Street, Baltimore.

I am keeping a list of these places, and shall be glad to be informed of others. The combination of instrument-makers can keep this steel if they wish; its cost is, I believe, about thirty-five cents per pound, and I am sure they will be glad to have us publish a list of places where it can be procured.

A remark has been made that the better a matrix fits the worse it is. In many cases there is danger in narrowing the orifice of the cavity. I saw Dr. Bogue using a flat piece of steel with a broken end of a gum-lancet for a wedge. I think he told me he knew of others who had used it at about the same time as himself. In one case I have used the wedge itself as a matrix. The flat steel has this advantage,—that the matrix fitting against the gum can, with this narrow wedge, be tipped back against the adjoining tooth, and the walls of the cavity are exposed to view and can be reached at all points by the plugger. The excess of gold is only at the edges of the matrix. I have made some wedges for this matrix, shaped like a knife-blade, except that they are more curved, and with the thicker end flattened like a spoon-handle, opposite from the blade of the wedge; this makes them easy to insert or remove with the fingers without the aid of pliers. They are easily made of differing lengths and thicknesses, of copper or German-silver wire. In some the flat end is curved back so as to rest against the gum, holding the rubber dam away from the tooth.

I would like to call attention again to the use of gutta-percha as a matrix, not only for filling with amalgam but with oxyphosphate, and especially for applications of medicine in treatment, and the ease with which a molar tooth can be converted into a simple tunnel with gutta-percha as a matrix. And also the safety with which arsenic can be applied,—putting on the rubber dam, varnishing the edges of the cavity and the adjoining tooth, using a soft, low grade of gutta-percha, making the application thin, and covering with cotton and another piece of gutta-percha. When the patient returns for removal of application, only the latter piece of gutta-percha need be removed, leaving the funnel intact, the arsenic or other application may be syringed out, and further treatment given if needed. The certainty of result, where medicine is in contact with the tooth without being diluted with the fluids of the mouth, is very much increased, and it has the added advantage that, as far as odor is an indication, we have it isolated from fermentation, and know that it comes entirely from the cavity, if there is any odor.

There is another advantage which the matrix has in the Buckland method of inserting amalgam. It is his practice to put oxyphosphate against the pulp wall in shallow cavities, and packing the amalgam against that, taking care of all the edges, and forcing some points of the amalgam into the soft cement. This method can be reversed with the matrix by packing the amalgam against it and against the side wall, then placing thin cement against the pulp wall and covering with amalgam. Results will be better if the matrix is left in place until the amalgam is hardened.

The gutta-percha matrix can be used for oxyphosphate, and, by covering the crown of the filling with gutta-percha after insertion, the filling can be protected from moisture as long as need be.

*Dr. Wilson.*—I have a matrix consisting of a thin band of steel; on the ends of the steel are two posts, one threaded and the other smooth, and it is tightened by a screw. I prefer it to any other matrix.

*Dr. Meriam.*—This thin, hard rubber, furnished by dealers in electrical supplies, makes a matrix that is very readily adapted. A little heat softens it so that it is readily cut with a pen-knife.

*Dr. G. T. Baker.*—I would like to present something which I have here that I have used for a matrix with a good deal of satisfaction. It is made of Dr. Parmly Brown's German-silver polishing strips and the Lee pull-back for a screw. A piece of silver, a little longer than will reach around the tooth, is cut off and a hole punched through both ends, through which the screw passes into the nut. It is then tightened with the key. I will pass it around.

*Dr. Stevens.*—I want to emphasize one thing that Dr. Baker said, and that is in regard to the durability of cement fillings at the cervical wall when the filling is properly inserted. I have made the assertion to some of you here that my experience was that cement fillings did not waste away at the cervical wall when they were properly inserted. Dr. Baker is right with respect to that point. I had a case the other day where a cement filling had been in the mouth six or seven years, and all that remained of the filling was at the cervical wall, and it has been my experience that a cement filling made with any good cement, and properly inserted, will last longer at the cervical wall than at any other point.

*Dr. Barker.*—As long ago as my student days I saw a matrix used. It consisted of the uncut and drawn tempered end of a separating file supported by wedging. This may have been the forerunner of that class of matrices of which Dr. Louis Jack's is a good type. Such matrices are available only when teeth are in contact. Where teeth are not in contact it is necessary that the matrix embrace the tooth to be operated upon, and not depend for stability on an adjoining tooth. To meet this want various loop and band matrices have been thrown on the market. One of them, which I procured of Dr. Coull, in Washington, I have used with reasonable satisfaction. It is essentially the same as the matrix Dr. Wilson has described to-night. In my own practice I have found that a matrix improvised at the chair is more valuable than one I can buy. My plan is to use a thin strip of copper, brass, or German silver having one side silver plated. I cut off a piece of the metal a little wider than the depth of the cavity, loop it about the tooth, and grasp the free ends with a pair of pliers, thereby bringing the matrix firmly about the tooth. The band is then removed and soldered with soft solder, after which it is slipped back onto the tooth. If you have done your work accurately, you will find your matrix fitting with reasonable firmness, but, if need be, you can then insert a simple wedge at the point where you have done your soldering and have a matrix, all things considered, much better than anything else I have seen used.

The difficulty with a matrix is to get it close to the cervical border. This can be accomplished by the use of burnishers. The best result is attained by preparing the cavity and filling it with gutta-percha, or some material of that kind, making the contour of just the form needed. Form the matrix to this and then remove temporary filling.

There is another subject of which I wish to speak,—and that is, the incidental matter which Dr. Baker brought up, and which Dr. Stevens has alluded to, concerning the dissolving out of zinc phosphate fillings. I supposed that I knew how to handle oxyphosphate, or zinc phosphate, as well as anybody. I have put in a good many such fillings, I think, intelligently and with care, following the directions as we get them at the dental depots. In spite of care, the zinc phosphate fillings, after a couple of years, have dissolved and disintegrated, whether by the action of alkalies or acids I do not know. I only speak of my own practice. I have ceased entirely to rely upon zinc phosphate filling at the cervical wall. I always use a gutta-percha there and build down with a zinc phosphate. If there is any principle or method by which we may use zinc phosphate fillings more effectually, I would like to know it. Dr. Stevens says they should be kept dry. Let me say that follows as a matter of course,—it ought to go without saying that we insert zinc phosphate fillings under conditions of dryness. I use the rubber dam almost everywhere, and allow not less than twenty minutes to half an hour, or even longer if I can get the time, for a zinc phosphate filling to harden,—and yet I get these results. I do not use the zinc phosphate in a creamy state, but in a condition of good consistent putty, so that the mix is reasonably firm, and allow it to set unmolested and undisturbed.

*Dr. Smith.*—I would like to ask the gentleman if he uses a matrix in putting in his zinc phosphate fillings?

*Dr. Barker.*—No, sir, I do not.

*Dr. Smith.*—By doing so you will find that you will get more solidity to the fillings.

*Dr. Barker.*—Do you claim that there is any efficacy in the material being put against a metallic surface?

*Dr. Smith.*—Not particularly, but you could pack the filling in so much more firmly.

*Dr. Meriam.*—I have never seen either oxyphosphate, oxychloride, or gutta-percha that did not dissolve in an acid mouth. The oxide of zinc itself is soluble in both acids and alkalies, and, in buccal cavities in the lower third molars, what is called the "rotting" of gutta-percha is really the dissolving from it of the oxide; and I question if, in this position, phosphate of zinc fillings would last even if put in with a matrix, assuming that the patient did not give increased care, and thus change the conditions. We should remember that the phosphate of zinc may be soluble in one mouth and not in another. I keep in my medicine-case a dilute acid, the "pickle" of gold workers, which I use when I am doing small soldering. After mixing my oxyphosphate on the ordinary porcelain slab, I wipe the adhering cement from the slab with a little of this acid on cotton. Of course we have no acid so strong as this in the mouth, but it shows that the cement is soluble in acid.

*Dr. Baker.*—My point is simply this: by using a matrix it will enable us to do what we cannot possibly do without it. We get a uniform solidity to the filling.

*Dr. Meriam.*—I will show a new root-canal drill that was also sent over by Mr. Brunton. This has come in a letter since he sent the matrices. There is nothing new about it, with the exception that it is made from piano-wire, and is supposed to give us a point that will not break in the roots. Mr. Brunton writes that it is to be mounted for the hand-piece. When I go to New York I shall give it to Mr. Schmidt, and any one who wishes can order them from him.

I have a letter here from Dr. J. Morgan Howe, who sends me one of his new cervical retainers and clamp. He writes me as follows:

"The point of originality, which must be protected from appropriation by any enterprising egoist, is the invention of the cervical dam-retainer, which is a face-piece, so shaped as to be capable of being bound to the tooth by a clamp, or by any other means (no matter what), retaining the dam above the cavity, and at the same time permitting access to it. If you will remove the clamp with the forceps (from the tooth sent you), leaving the face-piece *in situ*, held by a piece of floss silk, you will see, I think, that where teeth are not very near to their neighbors a face-piece could be bound to the tooth with thread or its equivalent, if it were found desirable to use such means instead of a clamp. The retainer or face-piece can be modified in shape to any extent required for various cases, and still retain its identity. In individual cases (especially molars) it might be found best to make a retainer somewhat U-shaped, and bind it by the free ends to the tooth with floss silk. Then apply the dam, using thin rubber.

William H. Potter, D.M.D.,
*Editor American Academy Dental Science.*

